# 

**Published:** 1914-06-27

**Authors:** 


### CONTENTS.

| | PAGE |
|---|---|
| **The Voluntary Hospitals and Grants in Aid ...** | 337 |
| **A Research Ward at St. Thomas's Hospital...** | 338 |
| **Hospital and Institutional News ... ... ...** | 339 |
| The Birthday Honours—Medical Men and the Colonial List—Royal Infirmary, Liverpool—Renewed Staff Trouble at Camberwell—The Medical Officers and the Committee—The Guardians' Decision—The Development of the Ward Fund—Voluntary Boarders at Registered Hospitals—The Economic Factor—Hospital Beds for the London Police—New Pathologist at the Chest Hospital—Tuberculosis Officers and Combination—Solidarity in Tuberculosis Work—Sudden Death of a Hospital Surgeon—Mental Deficiency Work in London—The Cremation Society—Death of a Veteran Hospital Chaplain—The Surplus Panel Fund and the London Insurance Committee—Glasgow Chairman's Fine Record—Pharmacists and the British Pharmacopœia—The Originator of Hospital Sunday—The Admission of Patients—Important Extension of London Mental Hospital Work — The Portnalls Estate—This Week's Drug Market—" The Hampshire Tuberculosis Officership.' | |
| **Hospital Plans and their Selection ... ...** | 345 |
| Right and Wrong Methods : A Case in Point. | |

[See over.]

### CONTENTS—(*continued*).

PAGE

**The Chamberlain Memorial** ... ... ... 346
Mr. Harcourt at the Albert Dock Hospital.

**The Hospital Conference at Newcastle** ... 347

Some Lessons and Suggestions.

**The British Hospitals Association** ... ... 348
Fifth Annual Conference at Newcastle-on-Tyne, June 18 to 20, 1914.
Some Hospitals and Municipal Institutions. By the Lord Mayor of Newcastle, Councillor JOHNSTONE WALLACE, J.P.
The Voluntary Hospital: On Its Trial. By G. H. HUME, M.D., F.R.C.S. Ed., D.C.L., Vice-President Royal Victoria Infirmary.
The Discussion.

**Buildings Contemplated and in Progress** ... 360

**The Profession of Medicine** ... ... ... 361

The New Pharmacopœia.

PAGE

**Gifts and Bequests** ... ... ... ... ... ... 361

**The Editor's Letter-Box** ... ... ... ... 362
Lord Knutsford and Sir Victor Horsley—Motor-car Cots—Competitive Designs at Burnley.

**Round the World** ... ... ... ... ... ... 364

Fellow-Workers and their Doings.

**The Institutional Worker** ... ... (Suppl.) 1
The Samaritan Fund: Its Administration and Scope.
The Bergonié Apparatus.
Questions for June.
Questions Invited.
Miscellaneous.
Employment and Training.
The Sick and in Need.

